# *They are all inter-related, like a cycle*: an exploratory qualitative study of climate change, chronic stressors and mental health among displaced populations on the Thailand-Myanmar border

**DOI:** 10.3389/fpubh.2026.1846043

**Published:** 2026-09-03

**Authors:** Gracia Fellmeth, May Myo Thwin, Jasper Ko Ko Aung, Suphak Nosten, Nan San Wai, François Nosten, Chaisiri Angkurawaranon, Emma Plugge, Rose McGready

**Affiliations:** 1 National Perinatal Epidemiology Unit, Nuffield Department of Women’s and Reproductive Health, University of Oxford, Oxford, United Kingdom; 2 Shoklo Malaria Research Unit, Mahidol-Oxford Tropical Medicine Research Unit, Faculty of Tropical Medicine, Mahidol University, Mae Sot, Thailand; 3 Centre for Tropical Medicine and Global Health, Nuffield Department of Medicine, University of Oxford, Oxford, United Kingdom; 4 Department of Family Medicine, Faculty of Medicine, Chiang Mai University, Chiang Mai, Thailand; 5 Global Health and Chronic Conditions Research Centre, Chiang Mai University, Chiang Mai, Thailand; 6 Population Health Sciences, University of Southampton, Southampton, United Kingdom

**Keywords:** climate change, mental health, migrant, refugee, stressors

## Abstract

**Introduction:**

Climate change is a significant global health threat with disproportionate impacts across the Global South and among marginalized populations. Migrants and refugees experience multiple intersecting vulnerabilities including conflict, poverty and limited access to healthcare that may increase vulnerability to climate-related health impacts. This study aimed to explore perceptions of climate change and its impacts on mental health among migrant communities on the Thailand-Myanmar border.

**Methods:**

We conducted an exploratory qualitative study using focus group discussions (FGDs). Participants were rural migrant populations including pregnant women, adolescents, agricultural workers and healthcare workers living in Tak Province, Thailand. FGDs were audio-recorded, translated and analyzed thematically.

**Results:**

A total of 58 participants took part across eight FGDs. Participants’ narratives centered around stressors they faced on account of their precarious legal status, financial insecurity and conflict-related trauma. Climate change was rarely raised spontaneously. Following direct prompts about climate change, participants described increasing exposure to extreme heat, flooding and environmental degradation which disrupted livelihoods and exacerbated existing hardship. The multiple stressors participants described interconnected to form a complex web of challenges.

**Discussion:**

In this conflict-affected, resource-constrained setting, climate change was rarely raised spontaneously as a life stressor. Against a backdrop of acute legal and economic stressors, climate-related distress may not be perceived as the most pressing concern. However, climate change may act as an indirect stressor through its impacts on livelihoods and exacerbation of structural precarity. Addressing the inter-related challenges of climate change, displacement, poverty and mental health requires integrative, climate-sensitive and migrant-inclusive health policies and care.

## Introduction

Climate change is widely recognized as one of the most significant emerging threats to health, with impacts disproportionately borne by settings in the Global South (1). The scale and pace of climate change are unprecedented, and evidence increasingly links climate-related hazards with adverse physical and psychological health outcomes (1, 2). As well as posing immediate threats to life, extreme weather events and environmental disruptions can increase the risk of psychological distress, anxiety, depression, post-traumatic stress disorder (PTSD) and suicide (3, 4). These mental health impacts may arise through direct exposure to rapid-onset hazards such as flooding, storms, heatwaves or smoke, as well as through slower-onset processes, including changing rainfall patterns, seasonal uncertainty and environmental degradation – all of which can in turn impact livelihoods and food security. Mental health impacts may also emerge through the anticipation of future harms and uncertainty about environmental change, sometimes described as ‘eco-anxiety’ (5). In this paper, we use the term ‘climate change’ to refer to the broader structural process of anthropogenic environmental change. We distinguish this from climate variability, environmental degradation and individuals’ perception-based accounts of environmental stress, recognizing that climate change is often experienced materially through local manifestations such as changes in work, food security, mobility and exposure to heat or other environmental hazards.

Displaced populations are particularly vulnerable to climate-related harms (6). Climate-related events and environmental stressors shape displacement, vulnerability and mental health through political, economic and historical conditions including conflict, insecure labor, poverty and limited access to healthcare (7, 8). In the Global South, and in conflict-affected settings especially, these dynamics can be understood through frameworks of structural violence, precarity and political ecology, which emphasize how unequal power relations shape exposure to environmental hazards and capacity to adapt (9). Many displaced populations already experience intersecting forms of disadvantage such as insecure housing and income, lack of legal documentation and limited social protection (10, 11). Those with precarious legal status are often employed in informal or irregular sectors with a lack of legal protections that render them more vulnerable to exterme working conditions (12). Within construction and agricultural sectors, for example, migrant workers may face prolonged heat exposure (12). Agricultural work and farming are particularly climate-sensitive, meaning that environmental variability can exacerbate instability of already insecure incomes (6, 13). These cumulative impacts of displacement, climate stressors and wider socio-political determinants place migrant populations at risk of poor mental health. The combined effects of climate change, migration and health have been described as syndemic to acknowledge their complex and mutually-reinforcing dynamics (11).

Much of the existing evidence on climate change to date stems from high-income settings, with comparatively little attention to displaced populations in resource-constrained contexts (13). Our study explored perceptions of climate change and mental health among migrant communities on the Thailand-Myanmar border. Decades of civil conflict in Myanmar have led to mass population displacement to the border region with Thailand, where migrants’ lives are shaped by legal insecurity, economic precarity and constrained access to services. This region has also experienced extreme weather events including cyclones and typhoons, environmental degradation and heatwaves in recent years (14). We aimed to explore migrant communities’ experiences and perceptions of climate change, identify the impacts of climate change on mental health and understand the perceived relative importance of climate change in the context of multiple stressors in this conflict-affected, resource-constrained setting.

## Methods

We conducted this exploratory qualitative study using a series of focus group discussions (FGD) with migrant communities living on the Thailand-Myanmar border.

### Setting

Civil conflict in Myanmar has resulted in mass population displacement from Myanmar into neighboring Thailand. Political instability and armed conflict intensified following a further military coup in 2021. In recent years, the emergence of illegal cyber-scam centers along the border with Thailand has generated further security and political challenges (15). Displaced populations reside within refugee camps or in urban and rural settlements along the border, frequently with informal legal status and limited access to healthcare, livelihoods and educational opportunities (16). The Shoklo Malaria Unit (SMRU) in Tak Province has provided humanitarian healthcare services and led research that has resulted in significant health improvements for the local population for over three decades. There are longstanding and high established trust between SMRU and the migrant communities it serves.

### Participants

Participants were displaced, undocumented migrants from Myanmar on the Thailand-Myanmar border in Tak Province, northwestern Thailand. To capture diverse views from within the migrant community, we invited individuals from the following groups to take part: pregnant women; adolescents; agricultural laborers; and healthcare workers (medical and nursing staff and community health workers providing care to the migrant community, and who are themselves members of the migrant community). Eligible individuals were approached face-to-face or by telephone in the week leading up to the study by SMRU staff and migrant health volunteers (*aw sor thaw*; community outreach workers who have longstanding and trusting relationships with local communities). Individuals who agreed to take part in FGDs were asked to attend a suitable community space in their locality on a scheduled date and time, forming a convenience sample of participants. All adult (>18 years) participants provided written informed consent. Adolescent participants provided written assent, and informed consent was collected from a guardian or responsible adult. Participants who were not able to provide a signature provided a thumbprint, and an independent witness was asked to countersign the form. Participants were informed that there was no obligation to take part and that they could withdraw at any time without needing to provide a reason. Participants were offered refreshments and 200 Thai Baht as an acknowledgement of thanks for their time and participation. Transportation costs incurred to attend the FGD were also reimbursed.

### Study procedures

FGDs were chosen as a research tool because they have previously worked well in this setting for sensitive topics and because of their potential for dialogical, collaborative and reflective processes among participants (17–19). We sought to understand perspectives of diverse groups and anticipated that FGDs would allow participants to build upon, question and refine one another’s contributions, generating insights into shared collective priorities. This interactive potential of FGDs was therefore central to our study design, enabling us to examine what individual participants thought and how collective views might be articulated, affirmed or challenged through group discussion (19).

FGDs were conducted in Burmese and facilitated by a female SMRU researcher from Myanmar who is fluent in English and Burmese (MMT). During the FGD, real-time interpretation of the Burmese discussion into English was carried out by a male SMRU social scientist from Myanmar fluent in Burmese and English (JKKA). Other members of the research team (GF, EP, RM) were present at the FGDs as observers and asked additional questions if they arose. FGDs followed a topic guide developed by the research team that was piloted before the study among SMRU staff and refined (Supplementary Information SI1). The facilitator began each FGD by explaining the purpose of the study, explaining that all discussions were confidential and ensuring all participants were happy to proceed. Participants and the study team introduced themselves. The interview guide began with open questions about participants’ day-to-day lives, differences between former and current living situations, and stressors and challenges experienced. The interview guide then moved on to ask about climate change, if it had not been raised spontaneously as a stressor. Printed photographs depicting local climate-related and environmental events such as a landslide, burning of crops, deforestation, flooding and heat were used as prompts to guide this part of the discussion (Supplementary Information SI2). Participants were asked to select photographs they felt represented an event they had either experienced or felt was a concern, stressor or priority. Participants were invited to share with the group their reasons for selecting the photograph and explain what it meant to them. FGDs were conducted in October 2025.

### Sample size

We aimed to conduct up to two FGDs of 6–8 participants each with pregnant women, adolescents, agricultural workers and healthcare workers. Data saturation was not formally assessed, but we anticipated that our target sample size would enable us to capture the key themes emerging from each of these population subgroups.

### Analysis

FGDs were audio-recorded using a digital voice recorder. During FGDs, one study team member (JKKA) interpreted discussions into English in real time. Three study team members (GF, EP, RM) sitting alongside the interpreter noted down the English interpretation word-for-word in real time. Study team members also observed and noted dialogical and collaborative processes within the groups including interactions between participants, group dynamics and non-verbal communication. After each FGD, the study team convened to share their perspectives. After all FGDs were complete, the study team discussed commonalities and differences observed between groups and compared observations on group dynamics.

Due to time and resource constraints, it was not possible to produce full Burmese-language transcripts. Instead, the following approach was used. First, the word-for-word English-language notes made by the three study team members during FGDs (based on the interpreter’s real time interpretation) were compared and reconciled to assess consistency and accuracy. Second, any gaps or inconsistencies in the notes were rectified by listening to the audio-recordings. The relevant sections of the Burmese-language audio were identified, and the spoken content was translated into English and added to or used to correct the English-language notes. Finally, during coding, key excerpts of interest were identified from these English-language records. The audio-recordings were then revisited, and Burmese-speaking members of the study team transcribed the selected excerpts verbatim in Burmese and subsequently translated these into English. This ensured that, for selected excerpts relating to key themes emerging from the participants’ narratives, the nuanced wording and meaning could be checked against the original Burmese and retained in the English translation.

Finalized transcripts were analyzed using thematic analysis following the stages outlined by Braun and Clarke (20). Two study team members (GF, EP) independently performed preliminary inductive analysis to identify notable features emerging from the narratives. These features were discussed in depth with the entire study team. The team collaboratively grouped and coded these features into potential themes, which were iteratively reviewed and refined. Discrepant voices within the transcripts were highlighted to ensure that the range of views held by participants were captured accurately. Transcripts were not returned to participants for comment or correction, and participants did not provide feedback on the findings. The data was managed and analyzed using Microsoft Word. This study is reported according to the Consolidated Criteria for Reporting Qualitative Research (COREQ) checklist (21).

### Reflexivity statement

As a study team we acknowledge the possibility of unconscious biases influencing the facilitation of FGDs and interpretation of findings. The multi-disciplinary study team consisted of clinical and research staff experienced in conducting qualitative research with displaced communities in this setting. The team included researchers from Myanmar who have themselves experienced displacement to the Thailand-Myanmar border. These researchers from Myanmar and the senior author (RM) have lived on the border area for several decades and have longstanding expertise in participatory research methods and community engagement. Although the research team did not have prior relationships with FGD participants, their close ties with the migrant community enabled strong trust and rapport to be built within FGDs. Two other researchers are based in the UK but have lived for 1 year on the border (GF) and collaborated with SMRU on multiple studies (GF, EP).

### Ethical approval

Ethical approval was granted by Chiang Mai University Ethics Committee (298/2025), Oxford University Tropical Research Ethics Committee (OxTREC 600-24) and the Tak Community Advisory Board (TCAB202505).

### Public and participant involvement and engagement

This study was the first initiative in the region to talk directly with communities about climate change and understand the priorities of displaced communities. As there was significant uncertainty about the subject matter in this marginalized population, this exploratory study forms a useful platform to engage public and patients moving forwards.

## Results

Eight FGDs were conducted in rural community settings in Tak Province. A total of 58 participants took part including pregnant women, adolescents, agricultural laborers and healthcare workers (Table 1). Participants were aged between 15 and 44 years. One participant had arrived in Thailand only 10 days prior while some of the younger participants were born in Thailand and had never visited their home villages in Myanmar. Most participants were of Rakhine or Karen ethnicity and the majority did not hold legal documentation. FGDs lasted between 45 and 90 minutes.

**Table 1:** Summary of FGDs and participants.

FGD	Location	Participants (*n*)
1	Pop Phra (SMRU outreach clinic)	Aw Sor Thaws (community health workers); duration in Thailand ranging from 5 years to since birth (*n* = 8).
2	Mawker Tai (SMRU clinic)	SMRU healthcare workers; duration in Thailand ranging from 3–30 years (*n* = 8).
3	Mae Jaroen (SMRU outreach clinic)	Female agricultural laborers; ages 23–40 years; duration in Thailand ranging from 10 days to since birth (*n* = 8).
4	Mae Jaroen (SMRU outreach clinic)	Adolescent girls; ages 16–17 years; duration in Thailand ranging from 3 to 5 years (*n* = 7).
5	Mae Ka Sa (SMRU outreach clinic)	Male agricultural workers; ages 19–31 years; duration in Thailand ranging between 5 months and since birth (*n* = 5).
6	Kilometer-18 (rural compound)	Pregnant women; ages 19–35 years; duration in Thailand between 1.5 years and since birth (*n* = 7).
7	Pa Khai Mai (community centre)	Adolescent boys and girls; ages 15–18 years; duration in Thailand ranging between 2 years and since birth (*n* = 8).
8	Ban-xx (village leader compound)	Male agricultural laborers; ages 19–44 years; duration in Thailand ranging between 3 months and 20 years (*n* = 7).

Although FGDs were facilitated in a way as to encourage dialogue among participants, we found that direct exchange between participants was relatively uncommon. Participants more often responded sequentially to the discussion topic than directly to one another, which may reflect local norms around politeness and avoidance of open disagreement. However, other forms of interaction between participants were observed. For example, disclosures of experiences or concerns by one participant frequently prompted others to share similar experiences, suggesting that the group format facilitated reflection and mutual legitimation of experiences. The use of photograph prompts was particularly effective in stimulating discussion of climate-related stressors. Compared with earlier parts of the FGDs, during which participants tended to speak in turn and address the facilitator directly, photograph prompts generated more animated exchange between participants. Participants spoke directly to one another, commenting on the images and comparing their individual experiences of events depicted.

**Figure 1:**
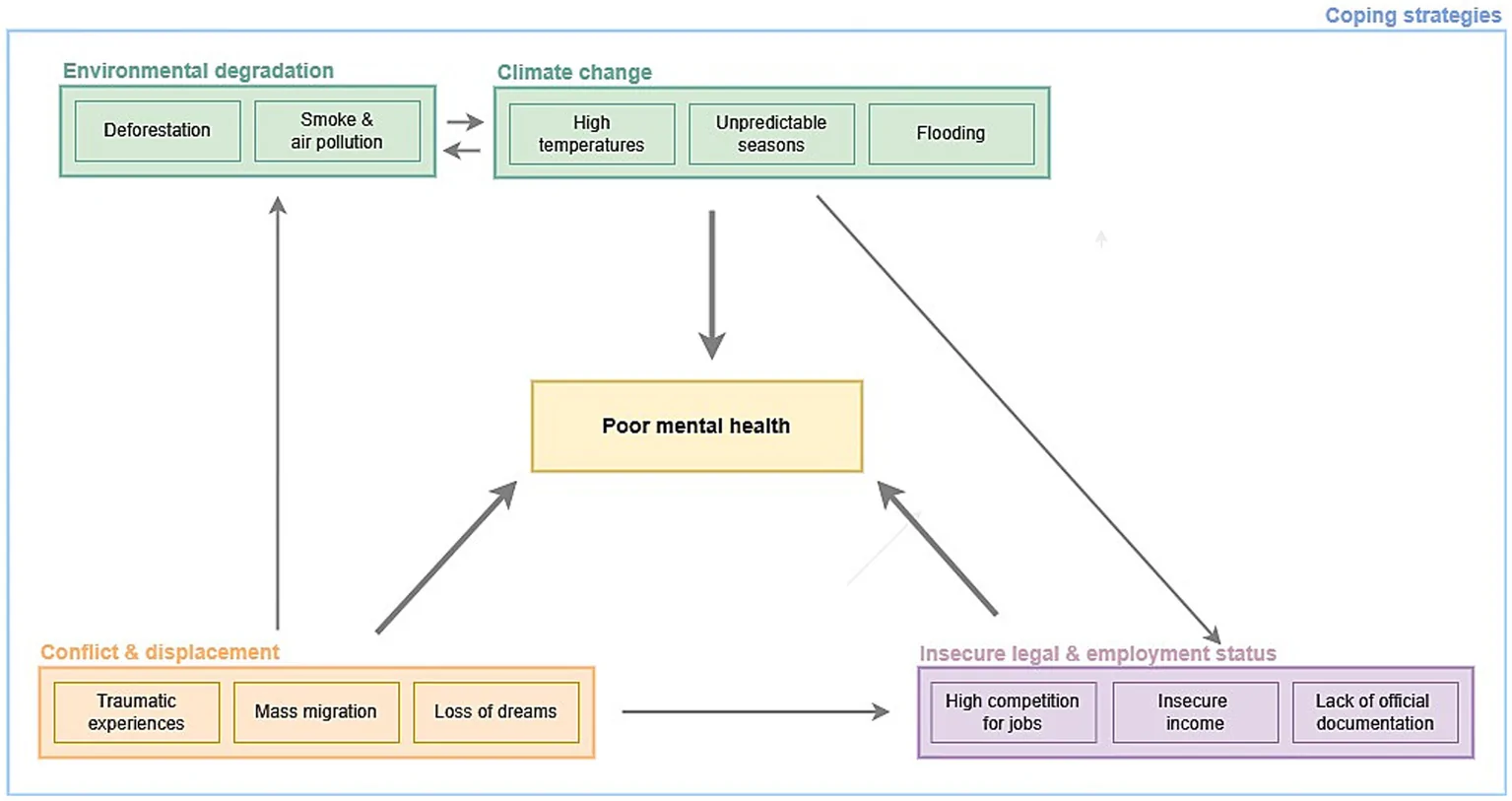
Summary of intersecting web of daily stressors and challenges experienced by participants.

Participants provided insights into their daily lives and the multitude of challenges they faced. Across all FGDs, the most prominent themes to emerge in response to the broad opening question about stressors and challenges were the lack of documentation and their resulting precarious legal status as migrants, and on difficulties making ends meet. Issues relating to climate change rarely arose spontaneously, instead only being discussed following specific question prompts about climate change. In response to these prompts, participants shared personal experiences of extreme weather events including flooding and heat and linked these to increasingly challenging working conditions. Participants did not always distinguish between climate change, seasonal variability and environmental degradation, but described these through experiences of heat, flooding, irregular rainfall, crop disruption and changing working conditions. A number of participants described the ways in which these stressors intersected, highlighting the complex web of challenges that characterize their lives. A simplified version of this web is shown in Figure 1; a more detailed version highlighting the complexity of mutually-reinforcing and intersecting stressors is shown in Supplementary Information SI3. This figure should not be interpreted as a causal pathway but as a representation of how legal precarity, conflict-related displacement, livelihood precarity and climate-related stressors reinforce one another and cumulatively contribute to mental distress. The emerging themes and subthemes are described below.

1. Legal and economic precarity as constant daily stressors

Participants described their everyday lives as being marked by constant stressors. The most commonly-described stressor was migrants’ lack of legal documentation or work permits, and their resulting precarious legal status in Thailand. This made it difficult to travel even within local areas due to the risk of fines and arrest. Participants reported that the number of police checkpoints and document controls had increased in recent years, making it increasingly difficult to access labor opportunities or seek healthcare.

*Before, there were not many checkpoints. If you spoke Thai, even just a little bit, you could pass, even without documents. But now there are many more [undocumented] migrants from Burma, and there are more checkpoints, more punishment and fines. (SMRU staff, FGD2)*

*Previously, traveling to the clinic was ok for pregnant women, but now, on the way to the clinic, there are many surprise checkpoints and full documentation checks. (Community health worker, FGD1)*

These restrictions on movement and living in constant fear of the police contributed to feelings of distress for many participants.

*The situation makes us feel very depressed because whenever we go anywhere, we always have to be afraid of the police. (Male agricultural worker, FGD5).*

*In the previous 5 years, I [could] move around more freely in my environment. Now, I need to inform my village leader each time, I need to pay for the transport, pay for the bike – it’s getting more and more strict. This affects us, it makes us more depressed. (Community health worker, FGD1).*

Along with the lack of legal documentation, participants described that it had become increasingly difficult to earn sufficient income to meet their daily needs. This was attributed to several inter-related factors. Increased migrant workers arriving at the border region as a result of conflict escalation in Myanmar meant there was increased competition for the limited daily jobs available, in turn contributing to a lowering of wages. At the same time, a broader cost of living crisis has resulted in higher food prices in recent years. Together, these factors meant that it was a constant challenge to earn enough to make ends meet.

*There are many working situation difficulties. More people are coming into this area. Last year, more people crossed and there are more coming in every year. At the same time the jobs are less and less, and the salary gets less and less. (Community health worker, FGD1)*

*The situation now is that we cannot save money. Even with two of us working, we cannot save anything. Our income and our outgoings are the same. Everything we make, we need to spend. When we were in Burma, even if we did not work for 1 week we did not need to worry about food because we grew some food ourselves to eat. But here, we worry a lot because if we do not find work, then we cannot eat. (Pregnant woman, FGD6)*

Participants described their willingness to work hard as being insufficient:

*We are really trying hard, and we really want to work, but the money we earn is not enough. (Male agricultural worker, FGD8)*

*I had to work like this [planting] before. Previously I could survive doing this. Now, I have to do double the work and still I have no money left over after paying for food. (Pregnant woman, FGD6)*

The constant pressure of having to work was also experienced by some of the younger participants who had left education in order to earn an income and help support their families. One of the adolescent participants had been working for several years:

*We know how to plant corn and cut sugarcane because we have been working for a long time. We cannot save money for our future or for our health because there is no regular work. There might be 1–2 days of work but then it stops, so the money is not enough. We would like to work in the factory, but because we are young and we do not have documents, we cannot. We have to wait until we are 18 years old. Before 18 years you cannot get a work permit. (Adolescent girl, FGD4)*

Low and irregular earnings also limited ability to enroll in the health insurance scheme for migrants:

*Now many people cannot buy health insurance because of the low daily income. I tried to enrol for 3–4 health insurance for my family but now I cannot afford MFund [health insurance scheme for migrants] for everyone in the family. (Female agricultural worker, FGD3)*

The pressures to find daily work and earn money to provide food for the family weighed on participants’ minds and impacted their mental health.

*We feel stressed because it’s not enough: food, school fees for our children, we do not have enough [money] and it’s a really depressing situation (Community health worker, FGD1)*

1. Political instability and conflict in Myanmar: past and ongoing traumas

The theme of political instability, war and conflict in Myanmar arose across many of the FGDs. The political situation was identified as the underlying cause of population displacement into Thailand:

*I came to Thailand because there is a war in my village. I cannot live there anymore so the whole family moved to Thailand. (Female agricultural labourer, FGD3)*

*If we had a good political situation in our country, then these things would not happen. The first thing that needs to be fixed is the political situation. (Community health worker, FGD1)*

Participants spoke of war-related traumas they had experienced. Some described traumas they had experienced in the distant past while others described more recent and even ongoing exposure to airstrikes in their villages and along the border area.

*I saw my house being burned down. (SMRU staff, FGD2)*

*One thing that I cannot forget is the time the Burmese army came to my village. I was with my mother at home. They came to the village and pointed the gun at me but luckily they did not shoot. We were really afraid so we ran from village to village until we reached Maela [refugee camp]. (SMRU staff, FGD2)*

*I try to communicate with my family in my home village but now there are airstrikes so we cannot communicate. There are airstrikes every day. (Female agricultural labourer, FGD3)*

*I was born in the refugee camp. My brother was killed by the army. My mother and I went back to Burma but because there was no education we came back. My mother is now in Karen State. I feel I need to be very careful. I feel not safe, afraid. There were airstrikes and bombing in 2023 – I still feel afraid now. (Male agricultural worker, FGD5)*

Worries around forced conscription into the Myanmar army were common among male participants, some of whom were unable to return to Myanmar because doing so would mean they had to join the army.

*The Burmese army came to my township for forced conscription. I escaped to here because I did not want to join the army. (Male agricultural worker, FGD5)*

1. Climate change and extreme weather events exacerbating lives that are already difficult

Following direct question prompts on climate change, participants described experiences of extreme weather events including flooding and heat, and also of air pollution from smoke from burning crops at the end of the harvest season. Participants described flooding, extreme heat and seasonal irregularity as occurring more frequently in recent years. Flooding – often attributed to deforestation – was associated with stress and disruption to livelihoods, work and education.

*I also experienced flooding last year. In my village, we are very close to the river. During most of the rainy season, the water level increases and there is flooding, so the houses close by have to flee because all of the houses will flood. (Male agricultural labourer, FGD8)*

*For this area, every year we face [flooding], but this year it happened more often, 2–3 times. Previously it was only 1 time per year. The farms are flooded. Ban-xx [village cluster] never flooded before, but this year it did. Because of this flooding, the schools had to close and there are no jobs. (Community health worker, FGD1)*

*People are chopping trees. There are forest fires and there is deforestation. Because of that, it encourages the flooding. This is why it’s happened 3 times this year. (SMRU staff, FGD2)*

Working in the extreme heat was physically challenging and made it difficult to work for a full day. Pregnant women described having to continue working despite very high temperatures. Some participants described having to shift to night-time working to avoid the heat or to finish the labor that could not be done in the heat of the day. Smoke from farm fires and extreme heat were associated with headaches, dizziness and breathing difficulties.

*Even when it’s so hot, we still have to do the work. This year, the temperature is getting higher and higher. I have to drink more water. I need to take more care than before. We have to rest often. (Female agricultural labourer, FGD3)*

*[There are] more farm fires and burning makes more smoke, and it makes things more hot. Because of this my father got sick very often. The smoke also gives me headache and cough. (Adolescent girl, FGD4).*

*When we go to work, the weather is really hot and humid, but we have to work in any condition, regardless. (Pregnant woman, FGD6)*

Participants also spoke about the changing and increasingly erratic weather patterns:

*Now we do not know if it’s rainy season or hot season – it’s changing (Community health worker, FGD1)*

*I have noticed that the climate has also changed. For 4 months over the summer now it is really hot. Everything is not like before. It is sunny, then humid, rainy, strong winds. It affects our health a lot. Many people get sick because of it. For example now, it’s already winter season but it’s still raining, and then some days it’s really hot. (Adolescent, FGD7)*

A common example of this unpredictability was that rain frequently continued beyond the normal duration of the rainy season, which then restricted labor opportunities:

*[The] weather now is changing – it is all mixed up. It’s not like it used to be with clear seasons. The beans and the corn – at the dry season it’s time to pick them and go to sell. But the rain does not stop, so we cannot pick them. (Female agricultural labourer, FGD3)*

*Picking chillis – if it’s raining, we cannot do it. The boss does not allow it; it must be dry weather when picking them. Therefore, we are [always] looking out for rain. While we are picking we do not eat, we do not go to the toilet, we are just rushing to finish before the rain starts.* (Community health worker, FGD1)

1. Intersectionality of challenges creating a complex web

Participants described the multiple challenges they face as intersecting and inter-related. Political instability in Myanmar, their own precarious legal status in Thailand, the limited employment opportunities available to them, low daily wages and competition for work were all compounded by changing weather patterns and increasingly common extreme weather events, forming a complex web of challenges shaping their lives.

*The people run away from the war, so they come here. Then here, there are floods so they have to leave again. (Community health worker, FGD1)*

*They are all inter-related, like a cycle. Because of the political situation, […] and because of the climate situation. Together, these cause the situation of joblessness. Going around in a circle like this. (Community health worker, FGD1)*

*Because of this deforestation there is landslides, earthquakes and flooding. The farms are also destroyed because of the flooding. Therefore, the market prices get more and more high. The migrant workers’ income is low, so they get into debt. (SMRU staff, FGD2)*

*When I think of the things around me, everything is being destroyed. There is fire, war, conflict. This makes me depressed. We’re only hearing about bad things, there are never any good things or developments. We feel very sad. Every day we hear the same situation. (SMRU staff, FGD2)*

Importantly, participants’ accounts suggest that climate-related stressors may influence mental health indirectly through their impact on labor, income security, debt and uncertainty. Flooding and rain reduces availability of agricultural labor; heat and smoke render physical labor more demanding; and legal precarity limited participants’ ability to move, seek alternative work or access support. In this way, climate-related and environmental stressors intensified the everyday conditions through which distress was produced.

1. Sadness, despair, loss of dreams and a deep longing to go home

Underlying participants’ narratives was a sense of sorrow and a longing to go home. Many individuals described missing their native homes and villages and family members who were still there. There was deep sadness and at times despair at the fact that they could not go back because of the dangers posed by conflict, war, conscription, loss of educational opportunities and the lack of employment opportunities in their own country. These themes were particularly prominent among the younger participants, many of whom became tearful when talking about how their lives had been before they migrated to Thailand, and when recounting their difficult current living situations.

*When I lived in Burma, it was not very difficult to work or survive. In our village I did not need to work. Just cleaning the house and helping, and then having fun. But here, it’s really difficult. We have to work and earn money. But because of the situation in Burma, we had to move. Actually, I would like to go back. (Adolescent girl, FGD4)*

*I came here to support my family. My vision is to return home. (Adolescent, FGD7)*

*Everyone wants to go back home. (Adolescent girl, FGD4)*

Many of the adolescent participants harbored hopes and aspirations for their futures:

*When I left Yangon, I had graduated from bakery school. I crossed the river and went to Mae Salit. I had a connection through my church and moved here to the migrant school. I got married last month. I am currently a daily worker on a farm. I was planning to work in a bakery but it is really difficult. I am very afraid: afraid of arrest. Otherwise, I would have liked to start my own restaurant. (Young male agricultural worker, FGD5)*

*I go to the school in this village but it only goes to the 8th standard [equivalent to 14 years old]. I would like to continue at school but I already reached the 8th standard so I cannot go any further. If I stayed in my home village in Burma I maybe could be going to college or university by now, but in Thailand I cannot. (Adolescent, FGD7).*

*My ambition is to be an educator, but my situation does not allow it [tearful]. (Adolescent girl, FGD4)*

1. Coping mechanisms

In some of the discussions, participants spoke about how they helped each other cope with their distress and sadness. Participants described how they attempted to endure and manage adversity in the context of multiple stressors. For most, coping was less about resolving structural conditions, and more about practical and emotional strategies that allowed them to ‘keep going’ despite poverty, legal insecurity, displacement and climate-related disruption.

Family relationships were a central source of resilience:

*I look at my children or my wife’s face. If I see my wife’s smiling face, then everything is fine. Even if I have no food to eat myself, I am fine. (Male agricultural worker, FGD8)*

Coping mechanisms included emotional release through crying, religious practice and efforts not to dwell on hardship. Participants described encouraging themselves by framing their hardship as temporary, which helped them to withstand difficult conditions and provided a reason to keep on going.

*Sometimes we cry. After crying, we feel refreshed. Sometimes we pray. Sometimes we keep our feelings inside and do not think too much about it. [We’re] just trying to take it day by day. We try to think: I have daily bread, I have a regular income – compared to others, things are good. We try to tell ourselves this. (SMRU staff, FGD2)*

*We encourage ourselves. We tell ourselves: it’s only temporary, it’s only like this for now. It will not always be like this. Life is difficult to get – we do not want to lose it. We think about our children. (Pregnant woman; FGD6)*

Taken together, participants’ coping strategies reveal resilience in the face of multiple hardships, showing how individuals sustain themselves emotionally and socially in circumstances where broader material and structural support is limited.

## Discussion

This exploratory study explored perceptions of climate change and mental health among displaced communities on the Thailand–Myanmar border. Our aim was to explore climate change against the backdrop of broader social, economic, legal and structural challenges faced by this population. Myanmar’s military coup of 2021 intensified a long-standing polycrisis of economic collapse, disruption of health and social services, food insecurity and significant humanitarian needs – all occurring alongside shrinking international aid budgets and increasing restrictions on humanitarian access (14). These conditions have taken a significant toll on mental health within Myanmar (22, 23). For displaced communities along the Thailand-Myanmar border, the coup has further strained already-limited livelihoods through currency depreciation, loss of employment opportunities, militarisation of rural areas and disruptions to cross-border mobility, all of which limit adaptive capacity to climate-related shocks such as flooding, extreme heat and crop failure.

We opened FGDs with broad questions to explore participants’ everyday lives and understand the main challenges faced. In response to these open questions, key stressors described spontaneously by participants were strikingly similar across all groups. Lack of legal documentation and difficulty making ends meet were consistently reported as the most pressing and pervasive stressors. Increasing migrant flows to the border area – resulting from continued conflict within Myanmar – has increased competition for jobs and driven down wages. Combined with increasing market prices, migrant communities struggle to get by, let alone save earnings for future needs: *“everything we make, we need to spend.”* Precarity of legal status compounded these financial challenges. Most of the participants were undocumented migrants, and the increasing number of police checkpoints was described as frightening and stressful. A key factor driving the tightening of security checks in recent years has been the emergence of cyber-scam centres on the Myanmar side of the border, in the immediate vicinity of where our FGDs took place (15). The constant threat of documentation checks severely restricted participants’ ability to take up labor opportunities beyond their immediate vicinity due to the fines associated with passing through a checkpoint without the correct documents, further limiting access to income opportunities and access to health clinics.

Climate change was rarely mentioned spontaneously in FGDs. This absence is in itself a notable finding about the perceived relative salience of climate change. It suggests that against a background of multiple economic and legal stressors, climate change may not be at the forefront of participants’ minds when they consider the challenges they face in their lives. Participants’ accounts of climate-related issues occurred in response to specific and direct questions, and it is important to acknowledge the framing effect of our prompts on participants’ narratives. Nevertheless, important insights were gained. Participants described experiences of changing climate patterns, extreme weather events and environmental degradation disrupting livelihoods and exacerbating existing hardships. Many participants had experienced flooding so severe that it required them to permanently leave their homes. Physically-demanding labor in extreme heat caused physical exhaustion and dizziness and led to some laborers opting to work at night, bringing its own risks to safety, especially for women. In recent years, heatwaves have struck Myanmar: in 2024, several towns in Myanmar were among the hottest places on earth with temperatures over 48 degrees Celsius and high numbers of heat-related deaths (14). Hot and rainy seasons are becoming increasingly unpredictable: participants described rainfall continuing beyond the usual rainy season, meaning that crops could not be harvested. Many participants were experienced farmers with strong insights into disturbances to seasonality in this tropical region of the world which previously had clearly delineated rainy and dry seasons.

While recognizing that these accounts arose following prompts, the observation that climate stressors were experienced not directly but indirectly, through impacts on income, food security, mobility and safety, is consistent with findings from other settings in the Global South suggesting that climate stress is often encountered materially through livelihood disruption and adaptation (24–26). In a study conducted with conflict-affected communities in Myanmar, perceptions of climate change were intertwined with experiences of conflict, insecurity, spiritual beliefs and sociopolitical disruption, and concerns around climate change were overshadowed by more pressing priorities of survival and security (27). Our findings similarly suggest that climate change may be materially present in everyday life even when it is not articulated as a separate or prioritized concern. More broadly, among migrant agricultural workers across regions in the Global South, occupational heat exposure, environmental degradation and drought have contributed to significant physical and psychological harm (8, 13, 28). For undocumented migrants with limited labor protections, these climate-related risks can be particularly acute. Therefore, although climate change was not at the forefront of participants’ narratives, climate-related issues are nevertheless embedded within participants’ everyday lives, making already-difficult living situations even more challenging.

The intersecting challenges migrant communities in this setting face are “*all inter-related, like a cycle.”* Unpredictable seasons disrupt agricultural livelihoods, intensifying income and food insecurity and causing stress. Exposure to extreme heat and smoke from burning crops is associated with physical symptoms including headaches, dizziness and respiratory problems, further limiting ability to work. These climate-related and environmental factors combine with the political situation in Myanmar, increasing migration flows to the border area and migrants’ precarious legal status in Thailand to form a complex web of challenges significantly impacting migrant communities’ mental health and ability to survive. The “web of challenges” figure represents these mutually-reinforcing stressors. Climate-related hazards contributed to distress both directly, through physical discomfort, perceived danger and uncertainty, and indirectly, through disruption to livelihoods, income and food security.

Importantly, these indirect pathways were not linear. Environmental disruption could reduce opportunities to work and agricultural productivity, resulting in income loss and food insecurity, increasing debt and uncertainty and intensifying pressure to work in hazardous conditions. In turn, poorer physical and mental health could further reduce capacity to work, reinforcing livelihood insecurity. Legal precarity and displacement constrained participants’ ability to adapt to these pressures by limiting movement and access to secure employment and healthcare, thereby shaping both exposure to environmental risks and the resources available to respond to them. The model helps to explain why participants often spoke about climate-related harms through the language of work, debt, mobility and uncertainty, rather than as isolated environmental concerns. In this regard, the effects of climate change were mediated and amplified by displacement, insecure labor, constrained mobility and limited material and legal resources.

Among adolescents, a prominent theme was the loss of dreams, hopes and aspirations. Adolescents spoke about their dreams of returning to school, completing higher education, becoming educators and opening their own restaurants and bakeries. They had been forced to put these dreams on hold in order to seek daily work to support their families. Participants described various coping strategies which often centered around support from peers, thinking about their families to keep themselves going and taking things *“day by day.”* However, these strategies are insufficient in the long-run to counteract the longstanding and cumulative burden of prolonged displacement and climate uncertainty, particularly among adolescents who expressed profound loss of future aspirations. A study of adolescent girls and young women in Myanmar reported on the significant toll the conflict has taken on this group, with almost half reporting direct experience of armed combat, one third being separated from family and an alarmingly high prevalence of depression and self-harm (16). More broadly, studies have highlighted adolescence as a particularly sensitive period for climate-related distress among displaced youth, compounding educational disruption and identity loss (4, 13). Our findings suggest adolescents are a key risk group in this setting.

Overall, our study suggests that climate change may compound social, legal, economic and structural challenges faced by forcibly displaced populations in resource-constrained settings. The novel contribution of this study lies in exploring how climate change is experienced against other significant stressors. Our findings suggest that climate change may be perceived not so much as a direct concern, but rather as a socially-mediated stressor operating through livelihood disruption, labor precarity, food insecurity and uncertainty about the future. This experience can be understood through the concept of environmental distress: distress arising from exposure to environmental hazards as well as from the erosion of livelihoods, security and expectations for the future (29). At the same time, participants’ vulnerability to these harms is influenced by conditions of precarity, in which insecure legal status, employment, income, housing and access to services reduce their capacity to absorb or adapt to environmental shocks.

These findings also illustrate how climate-related harms can intersect with structural and slow forms of violence. Structural violence draws attention to the political, legal and economic arrangements that unequally distribute exposure to risk and access to protection, while the concept of slow violence highlights cumulative, often less visible harms that unfold gradually through environmental degradation, livelihood erosion and repeated insecurity (30–32). Viewed in this way, climate change does not operate independently of displacement or marginalization but rather intensifies pre-existing inequalities and becomes incorporated into an accumulating burden of insecurity and distress (31, 33). Our findings support the notion of the climate-migration-health syndemic and the importance of adopting a ‘geopsychiatric’ lens through which to understand the broad, geopolitical determinants of mental health (8, 11, 34). Integrated policy responses addressing climate change and mental health among displaced populations and other vulnerable groups are urgently needed. Interventions must go beyond individual-level mental health support to address structural determinants – including legal protections, safe working conditions and access to healthcare – and ensure the development of integrative, interdisciplinary, climate-sensitive and migrant-inclusive health policy and care (8, 11).

### Strengths and limitations

This study has several strengths. The study team included representatives of the migrant community. FGDs were facilitated by a researcher from the Myanmar migrant community with extensive experience in qualitative methods, sensitive interviewing and community engagement. Alongside longstanding levels of trust between the migrant community and SMRU, this enabled strong rapport to be established within FGDs, fostering openness among the participants and a willingness to share personal experiences. Another strength is the diversity of participants, including men and women of different ages. Most participants were undocumented rural migrants, providing unique insights into the experiences of this group whose voices are often under-represented in research. The use of FGDs facilitated interaction between participants: when one participant voiced fears, worries or experiences that were emotionally difficult to disclose, others often followed by sharing similar concerns or elaborating on related experiences. Photograph prompts were particularly effective in stimulating inter-participant discussions, supporting more dialogical and reflexive engagement. Because these moments of inter-participant discussion involved overlapping speech among several participants, they were not always fully captured in the audio recordings or transcripts. We therefore draw on these observations cautiously to avoid making claims that exceed the available transcript data. However, overall we consider the use of FGDs a strength in that they generated data that reflected dynamic and reflexive processes among participants.

Limitations include the time-limited nature of data collection and the absence of verbatim transcription of FGDs in Burmese. Key themes were identified initially using the English-language translations, based on notes from real-time interpretation during FGDs. While we used audio-recordings to produce verbatim transcripts for key excerpts, it is possible that important themes were missed if these were not captured by the English-language notes. While including different population subgroups groups allowed us to hear a diversity of views, we may not have adequately captured nuanced differences. For example, evidence from other settings has suggested women are disproportionately impacted by climate change and that pregnant women experience particular challenges in relation to extreme heat. The limited number of pregnant women within our study may have meant that nuanced differences in experiences between different subgroups were not captured (13, 35). Finally, findings may not be generalizable beyond this context, though they offer transferable insights for similar displacement settings.

## Conclusion

Our findings suggest that among displaced communities on the Thailand-Myanmar border, climate change compounds the complex and intersecting web of social, political, economic and structural stressors impacting mental health. Income-insecurity, precarity of legal status and conflict were significant daily challenges experienced across diverse groups of migrant populations in this setting. While climate change was not identified spontaneously by participants as a key stressor, factors such as extreme heat and increasingly unpredictable seasons were recognized as exacerbating existing hardships. Climate-related distress was often mediated through everyday struggles over work, income, food insecurity and future uncertainty. Addressing mental health in this context requires intersectional, integrated responses that simultaneously address chronic precarity and build resilience to climate-related risks.

## Data Availability

The dataset presented in this article is not readily available because of the sensitive nature of the topic and participants’ precarious status. Requests to access the dataset should be directed to Professor Rose McGready (rose@shoklo-unit.com).
