# Supplementary material for: They are all inter-related, like a cycle: an exploratory qualitative study of climate change, chronic stressors and mental health among displaced populations on the Thailand-Myanmar border
Source: Front Public Health. 2026 Sep 3;14:1846043. doi: 10.3389/fpubh.2026.1846043 (PMC13582530; doi:10.3389/fpubh.2026.1846043)

Supplemental information (SI)

Table of contents

	Page
**SI1.** Focus group discussion (FGDs) topic guide	2
**SI2.** Examples of photographs used as prompts in FGDs	3
**SI3.** Detailed web of intersecting stressors and challenges experienced by participants	4

SI1. Focus group discussion (FGD) Topic Guide

Climate Change and Mental Health: Topic Guide for FGDs

- Introductions; explanations; ice-breakers

- Tell us about your life previously, as a child, growing up. What was your home [environment] like?

- What does your home look like now?
    - If appropriate: Are you living in a family home; tell us about your current living situation; who are you living with at the moment?
    - In what ways have things changed? How are things different from when you were growing up?

- What are some of the challenges you face?
- What are some of the stressors you face?
- What things in your life are hard for you?
- Are there things in your life that make you worry?

- Thinking about your work/labour, have there been changes in the conditions?
    - For example, changes in the type of work available, or how easy or difficult it is to do your work?
    - How do you cope? What has helped you cope with some of these changes?

Bring out picture prompts [flooding, landslide, heat, housing, smoke]

- Tell us about your experiences of any of these events.

- Thinking about some of these events, what do you think has brought about some of these changes?
- Have the changes had an impact on you / your families? In what ways? Have they impacted your wellbeing?

- If appropriate, further prompts about climate change:
    - What are your thoughts about climate change?
    - Is it something you think about?
    - Has it affected you or impacted your health or wellbeing?

- Are there any other issues you would like to share?
- Is there anything we have missed?

Summarise the outcome of the priorities discussion and ask if this is an accurate summary of what they have shared.

Give thanks.

SI2. Examples of photographs used as prompts in FGDs

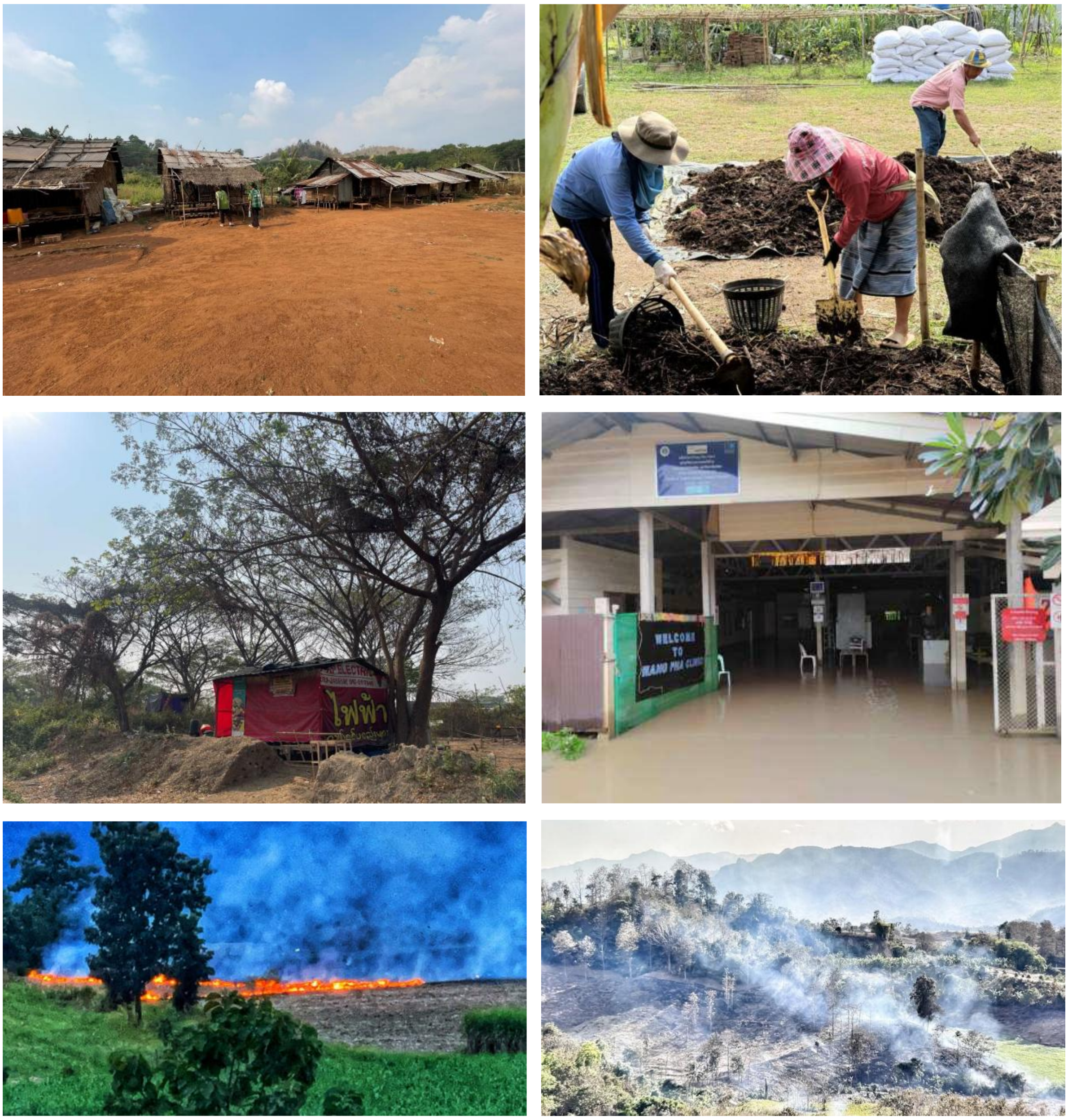

Copyright: Suphak Nosten

SI3. Detailed web of intersecting stressors and challenges experienced by participants

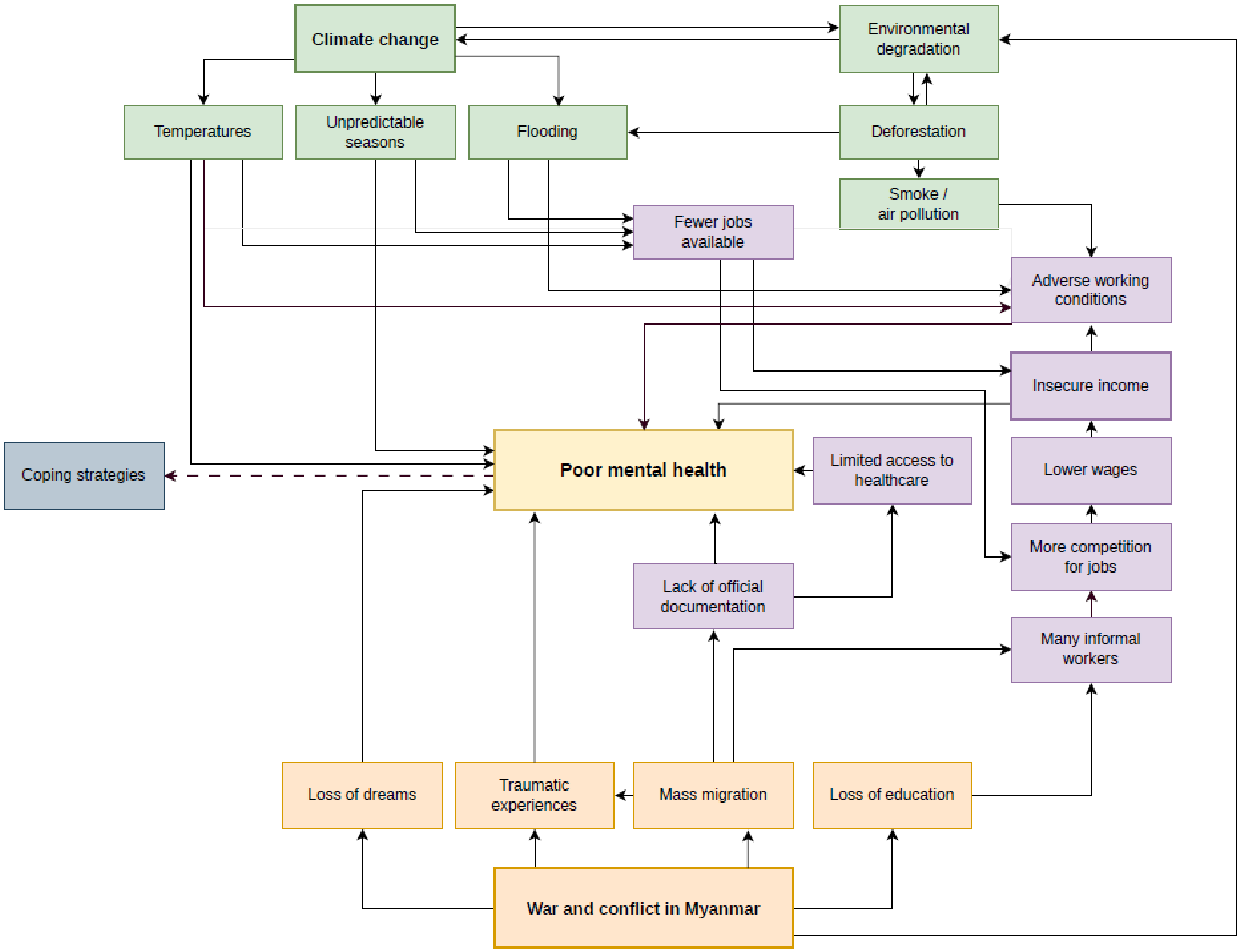

Supplement: Supplementary file 2 [file Data__Sheet__2.pdf]
